# Ultrasound-Based Deep Learning Radiomics Models for Predicting Primary and Secondary Salivary Gland Malignancies: A Multicenter Retrospective Study

**DOI:** 10.3390/bioengineering12040391

**Published:** 2025-04-05

**Authors:** Zhen Xia, Xiao-Chen Huang, Xin-Yu Xu, Qing Miao, Ming Wang, Meng-Jie Wu, Hao Zhang, Qi Jiang, Jing Zhuang, Qiang Wei, Wei Zhang

**Affiliations:** 1 Department of Ultrasound, Jiangsu Cancer Hospital & Jiangsu Institute of Cancer Research & The Affiliated Cancer Hospital of Nanjing Medical University, Nanjing 210009, China; xz_jsch@njmu.edu.cn (Z.X.); miaoqin0627@sina.com (Q.M.); zj_jsch@sina.com (J.Z.); 2 Department of Pathology, Jiangsu Cancer Hospital & Jiangsu Institute of Cancer Research & The Affiliated Cancer Hospital of Nanjing Medical University, Nanjing 210009, China; hxc3732353@njmu.edu.cn (X.-C.H.); xxy_jsch@sina.com (X.-Y.X.); 3 Department of Ultrasound, The 902nd Hospital of the Joint Logistics Support Force, Bengbu 233000, China; wm_902@sina.com; 4 Department of Ultrasound, The First Affiliated Hospital with Nanjing Medical University, Nanjing 210029, China; wmj_jssry@sina.com; 5 Department of Ultrasound, Affiliated Hospital of Nantong University, Nantong 226006, China; zh_ntfy@sina.com; 6 Department of Ultrasound, The Affiliated Jiangning Hospital of Nanjing Medical University, Nanjing 211100, China; jq_jnyy@sina.com

**Keywords:** salivary gland malignancies, ultrasound, radiomics, deep learning, interpretability

## Abstract

Background: Primary and secondary salivary gland malignancies differ significantly in treatment and prognosis. However, conventional ultrasonography often struggles to differentiate between these malignancies due to overlapping imaging features. We aimed to develop and evaluate noninvasive diagnostic models based on traditional ultrasound features, radiomics, and deep learning—independently or in combination—for distinguishing between primary and secondary salivary gland malignancies. Methods: This retrospective study included a total of 140 patients, comprising 68 with primary and 72 with secondary salivary gland malignancies, all pathologically confirmed, from four medical centers. Ultrasound features of salivary gland tumors were analyzed, and a radiomics model was established. Transfer learning with multiple pre-trained models was used to create deep learning (DL) models from which features were extracted and combined with radiomics features to construct a radiomics-deep learning (RadiomicsDL) model. A combined model was further developed by integrating ultrasound features. Least absolute shrinkage and selection operator (LASSO) regression and various machine learning algorithms were employed for feature selection and modeling. The optimal model was determined based on the area under the receiver operating characteristic curve (AUC), and interpretability was assessed using SHapley Additive exPlanations (SHAP). Results: The RadiomicsDL model, which combines radiomics and deep learning features using the Multi-Layer Perceptron (MLP), demonstrated the best performance on the test set with an AUC of 0.807. This surpassed the performances of the ultrasound (US), radiomics, DL, and combined models, which achieved AUCs of 0.421, 0.636, 0.763, and 0.711, respectively. SHAP analysis revealed that the radiomic feature Wavelet_LHH_glcm_SumEntropy contributed most significantly to the mode. Conclusions: The RadiomicsDL model based on ultrasound images provides an efficient and non-invasive method to differentiate between primary and secondary salivary gland malignancies.

## 1. Introduction

Salivary gland malignancies can be categorized into primary tumors, which include epithelial malignancies such as mucoepidermoid carcinoma and lymphomas represented by extranodal marginal zone B-cell lymphoma of mucosa-associated lymphoid tissue (MALT Lymphoma), and secondary tumors originating from metastases [1]. The distribution of secondary salivary gland malignancies varies regionally, with metastases from head and neck squamous cell carcinoma accounting for approximately 73%‒100% of cases [2,3]. Treatment and prognoses differ significantly between primary and secondary malignancies. While most patients undergo parotidectomy, neck lymph node dissection, or radiotherapy, the 5-year survival rate for secondary salivary gland squamous cell carcinoma remains substantially lower than that for primary malignancies (32.6% vs. 77.2%) [4]. Therefore, early identification of secondary salivary gland malignancies is crucial.

Although clinical data, imaging examinations, and fine needle aspiration (FNA) [5] can provide initial insights into whether a lesion is benign or malignant, accurate tumor classification still relies heavily on core needle biopsy or postoperative pathology. This reliance can lead to delayed diagnoses or wrong assessments, increasing surgical risks or resulting in missed treatment opportunities [6].

Ultrasound remains one of the preferred imaging modalities for salivary gland diseases. It is limited in the differential diagnosis of salivary gland tumors due to overlapping diagnostic features in the sonographic images [7]. While elastography and contrast-enhanced ultrasound techniques show promise, they have yet to be widely adopted [8]. Recently, radiomics and deep learning have demonstrated potential in the non-invasive differentiation of benign and malignant salivary gland tumors [9], as well as in distinguishing between different pathological subtypes of benign tumors [10,11,12].

However, no ultrasound-related studies have focused on the differentiation of secondary malignant salivary gland tumors, despite their significant proportion and the clear differences in treatment regimens compared to primary tumors. We aimed to develop a model integrating radiomics and deep learning using a retrospective analysis of ultrasound images from four major medical centers, providing a noninvasive approach to characterize salivary gland malignancies.

## 2. Materials and Methods

### 2.1. Patients

This retrospective study analyzed patients diagnosed with salivary gland malignancies across four centers in two regions. The inclusion criteria were as follows: patients with histopathologically confirmed salivary gland malignancies through surgical resection or biopsy, those who underwent preoperative ultrasound examination, and those with complete clinical data. The exclusion criteria were patients with poor-quality ultrasound images impeding accurate diagnosis, salivary gland tumors resulting from the invasion of adjacent malignant tumors, recurrent salivary gland malignancies following total resection, and cases where pathology could not definitively confirm whether malignancies were primary or secondary.

**Figure 1:**
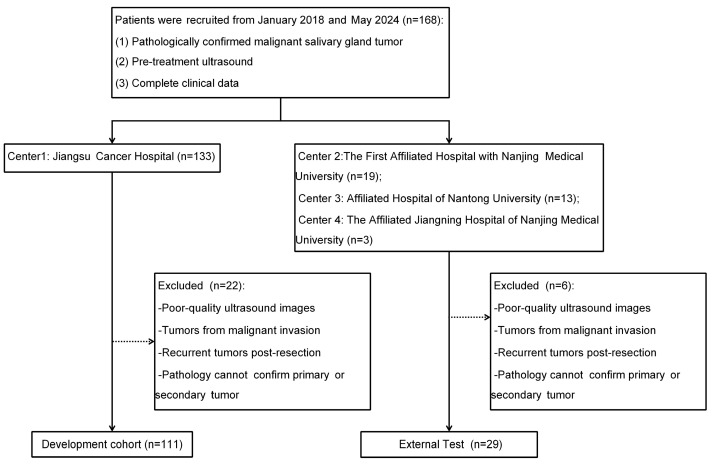
Flow diagram.

A total of 140 patients were ultimately enrolled, including 111 from Jiangsu Cancer Hospital, who were split in a 7:3 ratio into training and internal validation sets. An additional 29 patients from the First Affiliated Hospital of Nanjing Medical University, Affiliated Hospital of Nantong University, and the Affiliated Jiangning Hospital of Nanjing Medical University were used as the external test set. A flow diagram is shown in Figure 1. This study complied with the Declaration of Helsinki and was approved by the Ethics Committee of Jiangsu Cancer Hospital (No. KY-2024-057; Date: 1 July 2024). The requirement for individual consent was waived.

### 2.2. Histopathological Outcomes

Pathological diagnoses were based on the 5th Edition of the World Health Organization Classification of Head and Neck Tumors [13]. At Jiangsu Cancer Hospital, all pathological diagnoses were re-evaluated by a pathologist (X.-C.H.) with over 6 years of experience. This process involved reviewing original diagnoses and assessing patients’ clinical data to confirm whether the malignancies were primary or secondary. For the external test set, pathological and clinical data from three regional medical centers were collected by M.-J.W., H.Z., and Q.J. The final diagnoses were confirmed by X.-C.H. using consistent pathological and clinical criteria (Tables S1 and S2 in Supplementary Materials).

### 2.3. Ultrasound Imaging

Due to the retrospective nature of the study, all ultrasound images were exported in a PNG format from the ultrasound report systems and stored on a computer, which ensured high image quality. The ultrasound devices used in the study include Mylab Twice and MyLab 90 (Esaote, Genoa, Italy), HI VISION Preirus (HITACHI, Tokyo, Japan), S2000 (Siemens Healthineers, Erlangen, Germany), LOGIQ E20 (GE Healthcare, Chicago, IL, USA), and ALOKA ARIETTA 850 (FUJI, Tokyo, Japan), all equipped with linear high-frequency probes (with a frequency range of approximately 5 to 12 MHz). The ultrasound images were reviewed by M.W., an experienced ultrasound physician with over 15 years of expertise at a hospital comparable in clinical and diagnostic capabilities to the four participating centers, while being blinded to the pathological diagnosis. To assess lesion features, ultrasound evaluations were guided by the American College of Radiology (ACR) Thyroid Imaging Reporting and Data System (TI-RADS) [14], as no established diagnostic standards exist for salivary gland malignancies. Ultrasound features evaluated included composition, echogenicity, shape, aspect ratio, margin, calcification, and posterior acoustic characteristics.

### 2.4. Labeling

The ultrasound physician (Z.X.), with 6 years of experience, adhered to standardized procedures and was blinded to lesion pathology during annotation. Regions of interest (ROIs) were delineated using ITK-SNAP (version 4.0.2, www.itksnap.org) (accessed on 20 December 2024), encompassing the entire tumor mass while excluding non-tumorous surrounding tissues. The delineated ROIs were then exported to the Neuroimaging Informatics Technology Initiative (NIFTI) format for subsequent model training.

### 2.5. Radiomics Features Extraction

The PyRadiomics library was employed to extract radiomics features using a multistep approach. The analyzed image types included the original image, along with various transformed versions, including Wavelet, Square, SquareRoot, Logarithm, Exponential, and Gradient. The extracted features were categorized into three groups: geometry, intensity, and texture. Geometry features describe the two-dimensional shape characteristics of the tumor. Intensity features capture the first-order statistical distribution of voxel intensities within the ROI. Texture features capture patterns and higher-order spatial distributions of intensities, and are extracted using multiple methods, including the Gray level co-occurrence matrix (GLCM), Gray level dependence matrix (GLDM), Gray level run length matrix (GLRLM), Gray level size zone matrix (GLSZM), and Neighboring gray tone difference matrix (NGTDM). Additionally, for three-dimensional features, the third dimension was set to 1 to accommodate specific computational requirements.

### 2.6. Radiomics Features Selection

Feature selection was conducted using the following methods: (1) Z-score standardization was applied to remove scale effects across all features; (2) independent sample t-tests or Mann–Whitney U tests were used to calculate the *p*-values for all features between the primary and secondary tumor groups, and features with *p*-values less than 0.05 were retained for further analysis; (3) Spearman correlation analysis was used to remove redundant features. Features with a correlation coefficient greater than 0.9 were considered highly correlated, and only one feature from each pair was retained to reduce redundancy; and (4) The Least Absolute Shrinkage and Selection Operator (LASSO) regression algorithm, combined with five-fold cross-validation, was employed to further eliminate irrelevant features [15]. The final selected features were used for modeling.

### 2.7. Deep Learning Training

Several deep learning models with ImageNet pre-trained weights were used for training and validation [16]. The training, internal validation, and external test datasets were loaded based on their respective class labels and were normalized using the ImageNet standard. The models were trained with a stochastic gradient descent (SGD) optimizer with an initial learning rate of 0.01, a batch size of 32, and 50 epochs. During training, model performance was evaluated using metrics including accuracy, precision, recall, and F1 scores. Additionally, confusion matrices and receiver operating characteristic (ROC) curves were generated to assess classification performance further.

### 2.8. Radiomics-Deep Learning (RadiomicsDL) and Combined Models

Deep learning features were extracted from the global average pooling (avgpool) layer of the trained model. The classification layer was removed, and the avgpool output, which captured high-level image semantics, was used as the feature vector. The input data underwent forward propagation and the extracted features were organized into a matrix. Principal component analysis (PCA) was applied to reduce dimensionality while retaining key information. Compressed feature vectors were used for modeling, thereby improving the efficiency and performance.

Compressed deep learning features were combined with selected radiomics features, and key features were retained through dimensionality reduction, similar to the radiomics features selection. These were used to develop the RadiomicsDL model, which was then combined with the ultrasound features to create the combined model.

### 2.9. Machine Learning Modeling

Six classical machine learning models (Logistic Regression [LR], Support Vector Machine [SVM], Random Forest, eXtreme Gradient Boosting [XGBoost], Light Gradient Boosting Machine [LightGBM], and Multi-Layer Perceptron [MLP]) were used for modeling. After training, ROC curves were plotted to compare the area under the receiver operating characteristic curve (AUC) across the training, internal validation, and external test datasets. The model with the best performance on the external test dataset was selected as the final model.

### 2.10. Model Interpretability

Gradient-weighted Class Activation Mapping (Grad-CAM) and SHapley Additive exPlanations (SHAP) techniques were utilized to improve the interpretability of the DL and RadiomicsDL models.

Grad-CAM highlights the key regions that influence classification by generating heat maps from the gradients of the target class with respect to convolutional feature maps. Overlaying these heat maps on ultrasound images reveals areas critical to the model’s performance and identifies clinically relevant features [17].

SHAP quantifies the contribution of individual features to the predictions. Globally, it identifies dominant features, while locally explaining individual predictions by visualizing the direction and magnitude of contributions, further improving interpretability [18].

### 2.11. Software and Statistical Analysis

Statistical analyses were performed using Python (version 3.7) and R (version 3.6.1). Categorical variables were presented as frequency (n) and percentage (%), while continuous variables were presented as mean ± standard deviation (SD). Group differences were assessed with chi-square or Fisher’s exact test for categorical variables, and independent samples t-tests or Mann–Whitney U tests for continuous variables. Correlation analyses were performed using Pearson’s or Spearman’s coefficients, as appropriate. The diagnostic performance of the model was evaluated using the AUC, sensitivity, specificity, and accuracy. Statistical differences between model performances were tested using Delong’s test. Statistical significance was set at *p* < 0.05.

## 3. Results

### 3.1. Clinical Characteristics

A total of 140 patients with 242 ultrasound images were included in the analysis. Of these, 111 patients from Jiangsu Cancer Hospital served as the development cohort, contributing 187 images. The images were randomly divided into the training set (130 images) and internal validation set (57 images) in a 7:3 ratio. The remaining 55 images, sourced from three other centers, formed the external test cohort. To prevent data leakage, we ensured that images from the same patient were not included in different subsets. A comparison of baseline characteristics between the development and external test cohorts revealed no significant differences (Table 1).

### 3.2. Ultrasound Features

The ultrasound (US) features were compared across the three groups based on pathological type (Table 2). The analysis identified statistically significant differences in the aspect ratio and posterior echoes within the training set. Univariate and multivariate logistic regression analyses determined that an aspect ratio <1 and enhanced posterior echoes were independent risk factors (Table 3). These features were subsequently incorporated into a logistic regression model.

### 3.3. Radiomics Modeling

**Figure 2:**
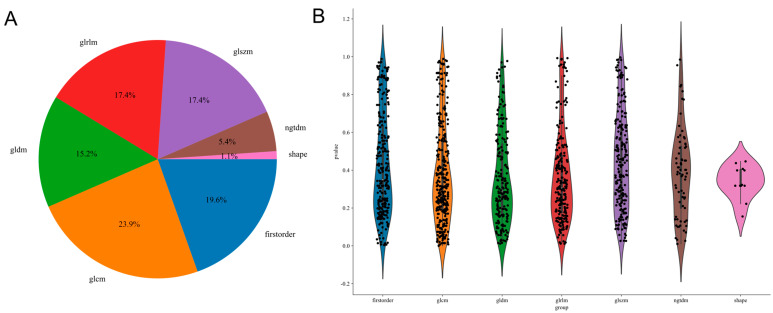
Radiomics features. (**A**) Proportions of the features. (**B**) Corresponding *p*−value results for the extracted features.

A total of 1288 radiomics features were extracted using PyRadiomics, including 252 firstorder features, 14 shape features, 308 GLCM, 196 GLDM, 224 GLRLM, 224 GLSZM, and 70 NGTDM features. All features were extracted using an in-house feature analysis program implemented in PyRadiomics (http://pyradiomics.readthedocs.io) (accessed on 20 December 2024). After normalization and statistical tests (*t*-tests or Mann–Whitney U tests) between the primary and secondary tumor groups, 55 statistically significant features were identified. Figure 2 shows all features and corresponding pvalue results. Spearman’s correlation analysis was applied, retaining 24 features by eliminating highly correlated pairs (r > 0.9). Using LASSO regression with five-fold cross-validation, the feature set was refined to 10 features with nonzero coefficients for subsequent modeling (Figure S1A–C in Supplementary Materials). Among the six machine learning algorithms evaluated, the SVM performed best, achieving AUC values of 0.841, 0.767, and 0.636 for the training, internal validation, and external test sets, respectively (Table 4).

### 3.4. Deep Learning Modeling

Eight commonly used ImageNet pre-trained models were systematically evaluated. Among these, Resnet50 demonstrated the best performance on the external test set and was selected for further analysis. The AUC values for the training, internal validation, and external test sets were 0.848, 0.746, and 0.763, respectively (Table 5).

### 3.5. Development of the RadiomicsDL Model with Integrated Features

A total of 2048 deep learning features were extracted from the average pooling layer of the trained DL model. These were reduced to 10 key components and combined with radiomics features. Through feature selection, three significant features were identified: DL_0, exponential_gldm_LargeDependenceHighGrayLevelEmphasis, and wavelet_LHH_glcm_SumEntropy (Figure S1D–F in Supplementary Materials). Machine-learning modeling revealed that the MLP model performed best. The resulting RadiomicsDL model achieved AUC values of 0.771 for the internal validation set and 0.807 for the external test set (Table 6).

### 3.6. Comparison of Model Performance

**Figure 3:**
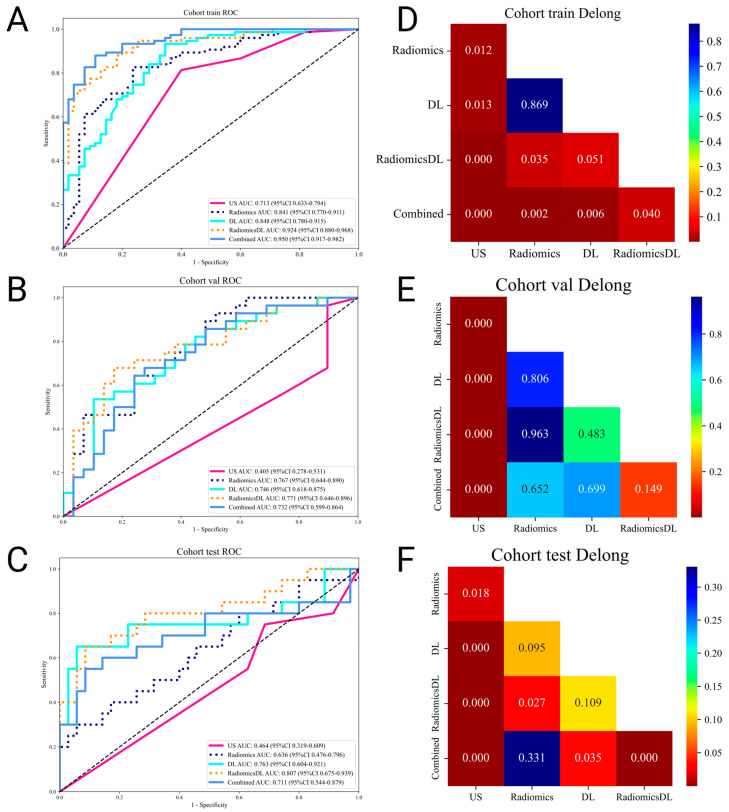
Comparison of ROC Curves and Delong Test results across training, validation, and test cohorts. (**A**–**C**) illustrate the ROC curves for the US, Radiomics, DL, RadiomicsDL, and combined models across training, validation, and test cohorts, respectively. (**D**–**F**) display the corresponding Delong test results as heat maps for each cohort, highlighting the statistical significance levels between the model comparisons. Color gradients represent *p*-values, with lower values indicating greater statistical significance.

The diagnostic performance of US, Radiomics, DL, RadiomicsDL, and combined models (integrating US and RadiomicsDL) was compared using receiver operating characteristic (ROC) curves and the DeLong test. The RadiomicsDL model achieved the highest AUC in the external test set (0.807), significantly outperforming the US and Radiomics models. However, no significant differences were found between the DL and combined models (Table 7, Figure 3).

### 3.7. Interpretability Analysis of DL and RadiomicsDL Models

**Figure 4:**
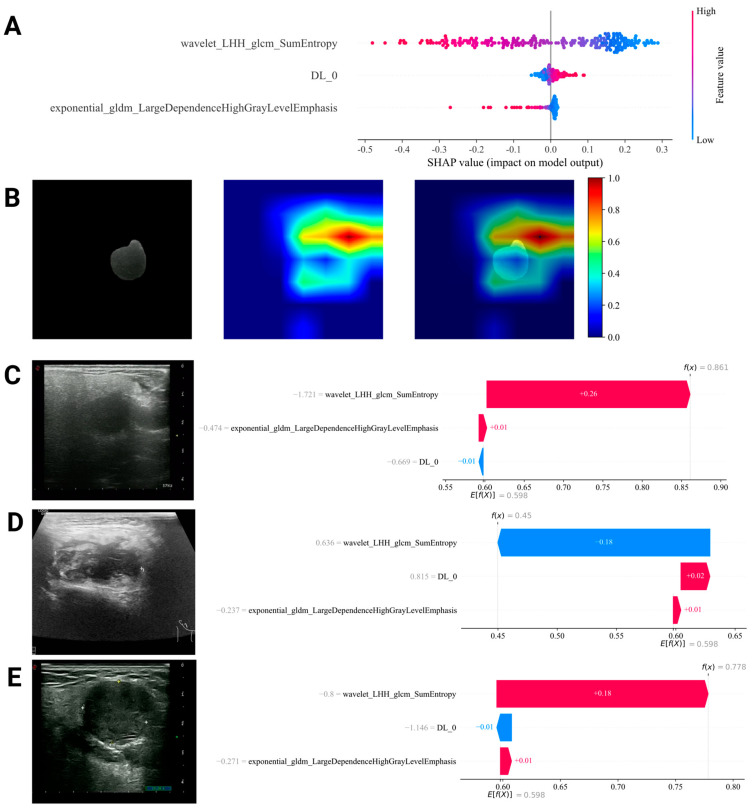
Model visualization. (**A**) SHAP summary plot: This plot demonstrates that the radiomic feature wavelet_LHH_glcm_SumEntropy has the highest impact on the predictions of the RadiomicsDL model. (**B**,**C**) Grad−CAM and SHAP diagram for a case of secondary salivary gland malignancy (metastatic adenocarcinoma). The DL model incorrectly classified this case as a primary tumor (**B**), with the Grad−CAM heatmap highlighting the tumor region predominantly in blue, suggesting the presence of a primary tumor, while red areas indicate the secondary tumor, which contributed to the misclassification. In contrast, the SHAP analysis of the RadiomicsDL model (**C**) supports the correct diagnosis as a metastatic lesion. (**D**) SHAP waterfall plot for a correctly classified case of primary salivary gland malignancy (diffuse large B−cell lymphoma). (**E**) SHAP waterfall plot for a correctly classified case of secondary salivary gland malignancy (metastatic small cell lung cancer).

SHAP interpretability was applied to the RadiomicsDL model, and the SHAP Summary Plot (Figure 4A) was generated. The results showed that, among the three features in the model (Wavelet_LHH_glcm_SumEntropy, Exponential_gldm_LargeDependenceHighGrayLevelEmphasis, and DL_0), the radiomic feature Wavelet_LHH_glcm_SumEntropy had the highest impact on the model’s predictions. Additionally, higher values of the two radiomics features were associated with a greater likelihood of primary tumors, whereas DL_0 showed the opposite relationship. SHAP waterfall plots, as shown in Figure 4C–E, are used to explain the prediction process of selected representative lesions. Grad-CAM heat maps were generated from the average pooling layer of the trained ResNet50 model to visualize the ROI in the ultrasound image (Figure 4B).

## 4. Discussion

In this study, radiomics and deep learning features were extracted from ultrasound images to develop predictive models, including US, Radiomics, DL, RadiomicsDL, and combined models. The models were externally validated across three independent central hospitals, demonstrating the robust diagnostic performance of the RadiomicsDL model. Notably, the RadiomicsDL model achieved an AUC of 0.807 in the external test dataset, effectively distinguishing between primary and secondary salivary gland malignancies. This result highlights the potential of RadiomicsDL for non-invasive clinical applications.

To the best of our knowledge, this is the first study to investigate the pathological classification of salivary gland malignancies using ultrasound imaging. In the training dataset, the aspect ratio and posterior echo were identified as statistically significant features distinguishing primary from secondary salivary gland malignancies. Specifically, an aspect ratio of <1 and posterior echo enhancement were independent indicators of secondary tumors. The US model, developed based on these features, achieved an AUC of 0.726 in the internal training set. However, its performance significantly declined in the external test dataset, with an AUC of only 0.421. This finding underscores the limitations of conventional ultrasound in accurately classifying malignant tumor subtypes. Similar challenges are reflected in its inconsistent sensitivity for distinguishing benign from malignant salivary gland tumors, previously reported to range from 38.9% to 88% [19]. Due to this limitation, the diagnostic performance of the combined model in the validation and test sets was hindered, which is also seen in the limitations of conventional ultrasound in the application to salivary gland tumors. The overlapping ultrasound characteristics among tumor types likely represent a key barrier to achieving higher predictive accuracy, highlighting the need for advanced diagnostic tools, such as radiomics or DL approaches, to improve pathological classification precision.

The Radiomics model achieved promising results in the training and internal validation datasets but showed signs of overfitting in the external test dataset. This finding suggests that, while radiomics models can achieve high accuracy within specific datasets, their generalizability and robustness across diverse datasets remain challenges. By contrast, the DL model demonstrated stable performance across all datasets, highlighting its ability to capture data complexity and adapt to heterogeneous data distributions. Previous studies have emphasized that integrating radiomics and DL features can enhance tumor differentiation, staging, and prognosis prediction compared with using either method alone [20,21]. This improvement was attributed to the multi-omics model incorporating additional critical parameters. The integration of radiomics and deep learning in the Radiomics_DL framework improved the AUC and resulted in optimal performance on both the internal test set and the external validation set. Compared to the standalone DL model, the Radiomics_DL model corrected several misclassifications, reducing the occurrence of false positives and false negatives (Figure 4). This further emphasizes the advantages of combining radiomics and deep learning, particularly in enhancing diagnostic accuracy.

In this study, we leveraged SHAP to analyze the interpretability of our proposed RadiomicsDL model, effectively visualizing the model’s evaluation process and prediction outcomes. The RadiomicsDL model was developed by integrating key deep learning features with selected radiomics features, combining two radiomics features and one deep learning-derived feature, with the SHAP summary plot identifying the radiomic feature Wavelet_LHH_glcm_SumEntropy as the most influential. This feature is derived from wavelet transform analysis of the GLCM. Our findings indicate that higher values of this feature correspond to an increased likelihood of primary tumor presence. This observation aligns with previous studies, which have demonstrated a correlation between this feature and favorable prognosis as well as reduced tumor invasiveness [22,23]. The integration of these two radiomics features with the deep learning-derived feature DL_0 significantly enhanced the model’s discriminative capability. Furthermore, leveraging SHAP for local interpretability analysis enables effective visualization of the model’s evaluation process and prediction outcomes.

Although this study provides encouraging preliminary results, it has several limitations. First, the retrospective design prevented the standardization of ultrasound image acquisition, and the analysis was limited to conventional ultrasound images, which may have constrained the model’s generalizability. Second, the relatively low incidence of salivary gland malignancies limited the sample size, despite cases being collected from multiple central hospitals. Variations in the regional distribution of pathological subtypes, potentially reflecting differences in population genetics or healthcare practices, may have introduced instability and reduced the reliability of the results. Moreover, since this study focused solely on binary classification of salivary gland malignancies, its applicability is limited. The failure to identify lymphomas separately is another limitation of this research.

To address these limitations, future studies should explore the integration of multimodal imaging data, such as adding Color Doppler Flow Imaging (CDFI) and elastography, to complement ultrasound findings and enhance diagnostic accuracy, particularly in further subtyping of tumors. Additionally, developing more generalized and versatile multilayer diagnostic models that can provide initial benign/malignant classification as well as further subtype classification for salivary gland tumors would be beneficial. Furthermore, collaborative efforts across multiple centers, along with the accumulation of large-scale datasets integrating clinical and genomic information, offer hope for building more comprehensive and robust diagnostic models.

## 5. Conclusions

In this study, we successfully extracted radiomics and deep learning features from salivary gland tumor ultrasound images. Through feature selection and machine learning, we developed a RadiomicsDL model capable of effectively distinguishing between primary and secondary salivary gland malignancies, thereby assisting clinicians in making accurate diagnoses.

## Figures and Tables

**Table 1:** Baseline characteristics of patients in the development and external test cohorts.

	Development Cohort (n = 111)	External Test Cohort (n = 29)	χ²	*p* Value
Age(y)			-	0.408
<50	21	3		
>50	90	26		
Sex			0.001	0.976
Male	64	16		
Female	47	13		
Location			0.018	0.893
Left	42	12		
Right	69	17		
Depth			0.163	0.687
Superficial	53	12		
Deep	58	17		
Pathology Type			2.030	0.154
Primary	50	18		
Secondary	61	11		

**Table 2:** Comparison of ultrasound features between primary and secondary salivary gland malignancies in training, validation, and test sets.

Feature Names	Train (n = 130)			Validation (n = 57)			Test (n = 55)		
	Primary	Secondary	*p* Value	Primary	Secondary	*p* Value	Primary	Secondary	*p* Value
Diameter (mm)	27.87 ± 12.39	26.18 ± 11.85	0.451	30.22 ± 19.15	27.70 ± 8.92	0.780	31.21 ± 8.27	23.63 ± 9.74	0.003
Composition			0.200			0.507			0.036
Solid	48 (87.27)	65 (86.67)		27 (93.10)	24 (85.71)		26 (74.29)	20 (100.00)	
cystic	2 (3.64)	0 (0.00)		0 (0.00)	1 (3.57)		0 (0.00)	0 (0.00)	
miscibility	5 (9.09)	10 (13.33)		2 (6.90)	3 (10.71)		9 (25.71)	0 (0.00)	
Echogenicity			0.220			0.986			0.003
Hyperecho	0 (0.00)	4 (5.33)		0 (0.00)	1 (3.57)		14 (40.00)	0 (0.00)	
Isoecho	55 (100.00)	71 (94.67)		29 (100.00)	27 (96.43)		20 (57.14)	20 (100.00)	
Hypoecho	0 (0.00)	0 (0.00)		0 (0.00)	0 (0.00)		1 (2.86)	0 (0.00)	
Aspect ratio			0.001			0.090			0.844
>1	22 (40.00)	10 (13.33)		3 (10.34)	9 (32.14)		11 (31.43)	5 (25.00)	
<1	33 (60.00)	65 (86.67)		26 (89.66)	19 (67.86)		24 (68.57)	15 (75.00)	
Shape			0.141			0.900			0.014
Regular	15 (27.27)	31 (41.33)		13 (44.83)	14 (50.00)		2 (5.71)	7 (35.00)	
Irregular	40 (72.73)	44 (58.67)		16 (55.17)	14 (50.00)		33 (94.29)	13 (65.00)	
Border			0.070			0.042			<0.001
Smooth	10 (18.18)	23 (30.67)		10 (34.48)	15 (53.57)		4 (11.43)	7 (35.00)	
Indistinct	18 (32.73)	21 (28.00)		7 (24.14)	1 (3.57)		7 (20.00)	11 (55.00)	
Lobulated or irregular	17 (30.91)	11 (14.67)		7 (24.14)	3 (10.71)		12 (34.29)	2 (10.00)	
Invasion	10 (18.18)	20 (26.67)		5 (17.24)	9 (32.14)		12 (34.29)	0 (0.00)	
Calcification			0.974			0.544			0.005
No	53 (96.36)	71 (94.67)		22 (75.86)	24 (85.71)		17 (48.57)	18 (90.00)	
Yes	2 (3.64)	4 (5.33)		7 (24.14)	4 (14.29)		18 (51.43)	2 (10.00)	
Posterior echoes			<0.001			0.504			0.048
Enhanced	34 (61.82)	70 (93.33)		22 (75.86)	24 (85.71)		30 (85.71)	12 (60.00)	
Non-enhanced	21 (21.82)	5 (4.00)		7 (6.90)	4 (3.57)		5 (2.86)	8	

**Table 3:** Univariate and multivariate analysis of ultrasound features in the training set.

Feature Names	OR_UNI	OR Lower 95%CI_UNI	OR Upper 95%CI_UNI	*p* Value _UNI	OR_MULTI	OR Lower 95%CI_MULTI	OR Upper 95%CI_MULTI	*p* Value _MULTI
Aspect ratio								
>1	Ref.				Ref.			
<1	4.333	1.839	10.209	0.001	3.688	1.478	9.204	0.005
Posterior echoes								
Enhanced	Ref.				Ref.			
Non-enhanced	0.116	0.040	0.333	<0.001	0.132	0.044	0.390	<0.001

UNI: Univariate Analysis; MULTI: Multivariate Analysis.

**Table 4:** Performance comparison of six machine learning models based on radiomics features.

Model	Accuracy	AUC	95% CI)	Sensitivity	Specificity	PPV	NPV	Precision	Recall	F1	Threshold
Train											
LR	0.700	0.787	0.709–0.866	0.587	0.855	0.846	0.603	0.846	0.587	0.693	0.627
SVM	0.792	0.841	0.770–0.911	0.813	0.764	0.824	0.750	0.824	0.813	0.819	0.599
RandomForest	0.846	0.931	0.891–0.972	0.893	0.782	0.848	0.843	0.848	0.893	0.870	0.543
XGBoost	0.938	0.988	0.974–1.000	0.933	0.945	0.959	0.912	0.959	0.933	0.946	0.618
LightGBM	0.862	0.900	0.843–0.957	0.840	0.891	0.913	0.803	0.913	0.840	0.875	0.575
MLP	0.723	0.756	0.673–0.840	0.867	0.527	0.714	0.744	0.714	0.867	0.783	0.534
Validation											
LR	0.719	0.756	0.624–0.888	0.893	0.552	0.658	0.842	0.658	0.893	0.758	0.459
SVM	0.684	0.767	0.644–0.890	0.893	0.483	0.625	0.824	0.625	0.893	0.735	0.516
RandomForest	0.719	0.757	0.630–0.885	0.964	0.483	0.643	0.933	0.643	0.964	0.771	0.460
XGBoost	0.719	0.763	0.637–0.889	0.821	0.621	0.676	0.783	0.676	0.821	0.742	0.535
LightGBM	0.684	0.722	0.584–0.860	0.607	0.759	0.708	0.667	0.708	0.607	0.654	0.629
MLP	0.684	0.672	0.527–0.818	0.964	0.414	0.614	0.923	0.614	0.964	0.75	0.510
Test											
LR	0.618	0.593	0.433–0.753	0.550	0.657	0.478	0.719	0.478	0.550	0.512	0.637
SVM	0.691	0.636	0.476–0.796	0.250	0.943	0.714	0.687	0.714	0.250	0.370	0.724
RandomForest	0.473	0.478	0.320–0.636	0.900	0.229	0.400	0.800	0.400	0.900	0.554	0.396
XGBoost	0.473	0.461	0.298–0.624	0.900	0.229	0.400	0.800	0.400	0.900	0.554	0.295
LightGBM	0.455	0.499	0.342–0.656	0.950	0.171	0.396	0.857	0.396	0.950	0.559	0.422
MLP	0.655	0.564	0.397–0.732	0.450	0.771	0.529	0.711	0.529	0.450	0.486	0.623

**Table 5:** Performance comparison of eight deep learning models.

Model	Accuracy	AUC	95% CI	Sensitivity	Specificity	PPV	NPV	Precision	Recall	F1	Threshold
Train											
densenet161	0.854	0.948	0.915–0.980	0.787	0.945	0.952	0.765	0.952	0.787	0.861	0.687
googlenet	0.715	0.776	0.697–0.856	0.733	0.691	0.764	0.655	0.764	0.733	0.748	0.578
inception_v3	0.692	0.784	0.707–0.862	0.573	0.855	0.843	0.595	0.843	0.573	0.683	0.673
resnet18	0.846	0.915	0.865–0.965	0.880	0.800	0.857	0.830	0.857	0.880	0.868	0.508
resnet50	0.808	0.848	0.780–0.915	0.920	0.655	0.784	0.857	0.784	0.920	0.847	0.340
resnet101	0.838	0.921	0.878–0.965	0.747	0.964	0.966	0.736	0.966	0.747	0.842	0.797
vgg11	0.623	0.661	0.569–0.754	0.400	0.927	0.882	0.531	0.882	0.400	0.550	0.592
ViT	0.523	0.424	0.324–0.524	0.693	0.291	0.571	0.410	0.571	0.693	0.627	0.505
Validation											
densenet161	0.667	0.687	0.545–0.829	0.929	0.414	0.605	0.857	0.605	0.929	0.732	0.185
googlenet	0.719	0.690	0.547–0.832	0.500	0.931	0.875	0.659	0.875	0.500	0.636	0.503
inception_v3	0.667	0.668	0.525–0.811	0.464	0.862	0.765	0.625	0.765	0.464	0.578	0.512
resnet18	0.702	0.748	0.621–0.876	0.679	0.724	0.704	0.700	0.704	0.679	0.691	0.233
resnet50	0.702	0.746	0.617–0.875	0.500	0.897	0.824	0.650	0.824	0.500	0.622	0.561
resnet101	0.737	0.775	0.649–0.901	0.714	0.759	0.741	0.733	0.741	0.714	0.727	0.290
vgg11	0.526	0.476	0.322–0.630	0.964	0.103	0.509	0.750	0.509	0.964	0.667	0.408
ViT	0.526	0.582	0.490–0.674	0.036	1.000	1.000	0.518	1.000	0.036	0.069	0.003
Test											
densenet161	0.709	0.761	0.622–0.901	0.750	0.686	0.577	0.828	0.577	0.750	0.652	0.320
googlenet	0.673	0.746	0.612–0.879	0.850	0.571	0.531	0.870	0.531	0.850	0.654	0.370
inception_v3	0.727	0.688	0.528–0.847	0.550	0.829	0.647	0.763	0.647	0.550	0.595	0.535
resnet18	0.727	0.704	0.553–0.855	0.450	0.886	0.692	0.738	0.692	0.450	0.545	0.423
resnet50	0.818	0.763	0.604–0.921	0.600	0.943	0.857	0.805	0.857	0.600	0.706	0.418
resnet101	0.709	0.729	0.586–0.871	0.700	0.714	0.583	0.806	0.583	0.700	0.636	0.300
vgg11	0.709	0.739	0.599–0.878	0.500	0.829	0.625	0.744	0.625	0.500	0.556	0.562
ViT	0.636	0.500	1.000–1.000	0.000	1.000	0.000	0.636	0.000	0.000	NaN	0.003

**Table 6:** Performance of six machine learning models based on radiomics and deep learning features.

Model	Accuracy	AUC	95% CI	Sensitivity	Specificity	PPV	NPV	Precision	Recall	F1	Threshold
Train											
LR	0.854	0.924	0.881–0.968	0.907	0.782	0.850	0.860	0.850	0.907	0.877	0.436
SVM	0.877	0.930	0.885–0.976	0.947	0.782	0.855	0.915	0.855	0.947	0.899	0.365
RandomForest	0.900	0.962	0.933–0.992	0.893	0.909	0.931	0.862	0.931	0.893	0.912	0.603
XGBoost	0.915	0.976	0.954–0.997	0.893	0.945	0.957	0.867	0.957	0.893	0.924	0.591
LightGBM	0.831	0.932	0.893–0.972	0.813	0.855	0.884	0.770	0.884	0.813	0.847	0.591
MLP	0.846	0.924	0.880–0.968	0.867	0.818	0.867	0.818	0.867	0.867	0.867	0.572
Validation											
LR	0.719	0.762	0.635–0.890	0.714	0.724	0.714	0.724	0.714	0.714	0.714	0.652
SVM	0.702	0.759	0.633–0.885	0.571	0.828	0.762	0.667	0.762	0.571	0.653	0.809
RandomForest	0.684	0.739	0.610–0.868	0.536	0.828	0.750	0.649	0.750	0.536	0.625	0.728
XGBoost	0.719	0.757	0.632–0.882	0.750	0.690	0.700	0.741	0.700	0.750	0.724	0.632
LightGBM	0.667	0.717	0.583–0.852	0.750	0.586	0.636	0.708	0.636	0.75	0.689	0.591
MLP	0.737	0.771	0.646–0.896	0.643	0.828	0.783	0.706	0.783	0.643	0.706	0.696
Test											
LR	0.727	0.734	0.589–0.879	0.600	0.800	0.632	0.778	0.632	0.600	0.615	0.901
SVM	0.745	0.741	0.594–0.889	0.600	0.829	0.667	0.784	0.667	0.600	0.632	0.884
RandomForest	0.727	0.786	0.664–0.907	0.650	0.771	0.619	0.794	0.619	0.650	0.634	0.714
XGBoost	0.764	0.754	0.614–0.894	0.550	0.886	0.733	0.775	0.733	0.550	0.629	0.945
LightGBM	0.727	0.731	0.583–0.878	0.700	0.743	0.609	0.812	0.609	0.700	0.651	0.630
MLP	0.800	0.807	0.675–0.939	0.600	0.914	0.800	0.800	0.800	0.600	0.686	0.796

**Table 7:** Performance of five models on the training, validation, and test sets.

Model	Accuracy	AUC	95% CI	Sensitivity	Specificity	PPV	NPV	Precision	Recall	F1	Threshold
Train											
US	0.423	0.726	0.646–0.806	0.000	1.000	0.000	0.423	0.000	0.000	NaN	0.735
Radiomics	0.792	0.841	0.770–0.911	0.813	0.764	0.824	0.750	0.824	0.813	0.819	0.599
DL	0.808	0.848	0.780–0.915	0.920	0.655	0.784	0.857	0.784	0.920	0.847	0.340
RadiomicsDL	0.846	0.924	0.880–0.968	0.867	0.818	0.867	0.818	0.867	0.867	0.867	0.572
Combined	0.877	0.950	0.917–0.982	0.867	0.891	0.915	0.831	0.915	0.867	0.890	0.547
Validation											
US	0.404	0.444	0.314–0.574	0.571	0.241	0.421	0.368	0.421	0.571	0.485	0.429
Radiomics	0.684	0.767	0.644–0.890	0.893	0.483	0.625	0.824	0.625	0.893	0.735	0.516
DL	0.702	0.746	0.618–0.875	0.500	0.897	0.824	0.650	0.824	0.500	0.622	0.561
RadiomicsDL	0.737	0.771	0.646–0.896	0.643	0.828	0.783	0.706	0.783	0.643	0.706	0.696
Combined	0.684	0.732	0.599–0.864	0.643	0.724	0.692	0.677	0.692	0.643	0.667	0.880
Test											
US	0.345	0.421	0.271–0.571	0.800	0.086	0.333	0.429	0.333	0.800	0.471	0.090
Radiomics	0.691	0.636	0.476–0.796	0.250	0.943	0.714	0.687	0.714	0.250	0.370	0.724
DL	0.818	0.763	0.604–0.921	0.600	0.943	0.857	0.805	0.857	0.600	0.706	0.418
RadiomicsDL	0.800	0.807	0.675–0.939	0.600	0.914	0.800	0.800	0.800	0.600	0.686	0.796
Combined	0.764	0.711	0.544–0.879	0.500	0.914	0.769	0.762	0.769	0.500	0.606	0.977

## Data Availability

The data presented in this study are available on request from the corresponding author (W.Z.) due to privacy restrictions.

## Supplementary Materials

The following supporting information can be downloaded at: https://www.mdpi.com/article/10.3390/bioengineering12040391/s1, Figure S1: Feature Selection Process for Radiomics and RadiomicsDL; Table S1: Distribution of Primary Malignancy Types Across Different Centers; Table S2: Distribution of Primary Sites of Secondary Malignancies Across Different Centers.

## Abbreviations

The following abbreviations are used in this manuscript:

DL	Deep learning
RadiomicsDL	Radiomics-deep learning
LASSO	Least absolute shrinkage and selection operator
AUC	Area under the receiver operating characteristic curve
SHAP	SHapley Additive exPlanations
MLP	Multi-Layer Perceptron
US	Ultrasound
MALT Lymphoma	Extranodal marginal zone B-cell lymphoma of mucosa-associated lymphoid tissue
FNA	Fine needle aspiration
ACR	American College of Radiology
TI-RADS	Thyroid Imaging Reporting and Data System
ROIs	Regions of interest
NIFTI	Neuroimaging Informatics Technology Initiative
GLCM	Gray level cooccurrence matrix
GLDM	Gray level dependence matrix
GLRLM	Gray level run length matrix
GLSZM	Gray level size zone matrix
NGTDM	Neighboring gray tone difference matrix
SGD	Stochastic gradient descent
ROC	Receiver operating characteristic
Avgpool	Average pooling
PCA	Principal component analysis
LR	Logistic Regression
SVM	Support Vector Machine
XGBoost	eXtreme Gradient Boosting
LightGBM	Light Gradient Boosting Machine
Grad-CAM	Gradient-weighted Class Activation Mapping
SD	Standard deviation
CDFI	Color Doppler Flow Imaging
