# Supplementary material for: Ultrasound-Based Deep Learning Radiomics Models for Predicting Primary and Secondary Salivary Gland Malignancies: A Multicenter Retrospective Study
Source: Bioengineering (Basel). 2025 Apr 5;12(4):391. doi: 10.3390/bioengineering12040391 (PMC12024528; doi:10.3390/bioengineering12040391)
Supplement: Supplementary file 1 [file bioengineering-12-00391-s001.zip › bioengineering-3474556-supplementary.pdf]

Table S1. Distribution of Primary Malignancy Types Across Different Centers

	Center 1 (n=50)	Center 2 (n=15)	Center 3 (n=2)	Center 4 (n=1)
Primary epithelial malignancies				
Mucoepidermoid carcinoma	11	0	0	0
Adenoid cystic carcinoma	5	1	1	1
Acinic cell carcinoma	6	0	0	0
Salivary duct carcinoma	4	1	0	0
Other Types	11	3	0	0
Lymphoma				
DLBCL	5	5	0	0
MALT Lymphoma	1	1	0	0
Burkitt Lymphoma	1	1	0	0
Other Types	6	3	1	0

Center 1: Jiangsu Cancer Hospital; Center 2: The First Affiliated Hospital with Nanjing Medical University; Center 3: Affiliated Hospital of Nantong University; Center 4: The Affiliated Jiangning Hospital of Nanjing Medical University; DLBCL: Diffuse Large B-cell Lymphoma; MALT Lymphoma: extranodal marginal zone B-cell lymphoma of mucosa-associated lymphoid tissue

Table S2. Distribution of Primary Sites of Secondary Malignancies Across Different Centers

	Center 1 (n=61)	Center 2 (n=1)	Center 3 (n=8)	Center 4 (n=2)
Nasopharynx	37	0	5	2
Lung	3	0	0	0
Malignant Melanoma	2	0	0	0
Thyroid	1	1	0	0
Unknown Origin	9	0	2	0
Others	9	0	1	0

Center 1: Jiangsu Cancer Hospital; Center 2: The First Affiliated Hospital with Nanjing Medical University; Center 3: Affiliated Hospital of Nantong University; Center 4: The Affiliated Jiangning Hospital of Nanjing Medical University

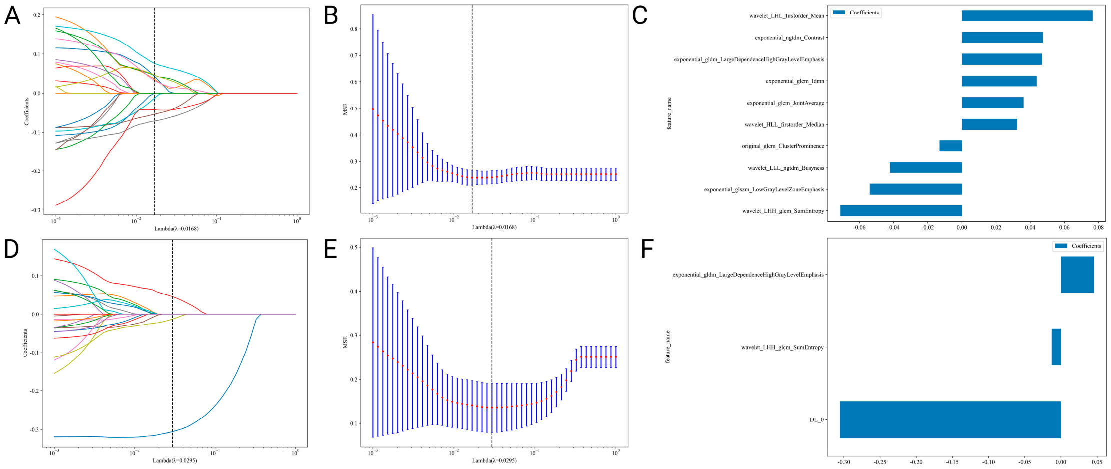

Figure S1. Feature Selection Process for Radiomics and RadiomicsDL

A-C: LASSO Coefficient Path Plot, LASSO Regression Cross-Validation Curve, and Feature Importance Plot in Radiomics Feature Selection; D-F: LASSO Coefficient Path Plot, LASSO Regression Cross-Validation Curve, and Feature Importance Plot in RadiomicsDL Feature Selection
